# Free Feeding of CpG-Oligodeoxynucleotide Particles Prophylactically Attenuates Allergic Airway Inflammation and Hyperresponsiveness in Mice

**DOI:** 10.3389/fimmu.2021.738041

**Published:** 2021-11-19

**Authors:** Takuma Okajima, Suguru Shigemori, Fu Namai, Tasuku Ogita, Takashi Sato, Takeshi Shimosato

**Affiliations:** Department of Biomolecular Innovation, Institute for Biomedical Sciences, Shinshu University, Nagano, Japan

**Keywords:** CpG-ODNs, oral delivery, mouse model of allergic asthma, airway inflammation, airway hyperresponsiveness, pulmonary vein cardiomyocytes, Reg3γ, gut microbiota

## Abstract

CpG-oligodeoxynucleotides (CpG-ODNs) constitute an attractive alternative for asthma treatment. However, very little evidence is available from studies on the oral administration of CpG-ODNs in animals. Previously, we developed acid-resistant particles (named ODNcap) as an oral delivery device for ODNs. Here, we showed that free feeding of an ODNcap-containing feed prophylactically attenuates allergic airway inflammation, hyperresponsiveness, and goblet cell hyperplasia in an ovalbumin-induced asthma model. Using transcriptomics-driven approaches, we demonstrated that injury of pulmonary vein cardiomyocytes accompanies allergen inhalation challenge, but is inhibited by ODNcap feeding. We also showed the participation of an airway antimicrobial peptide (Reg3γ) and fecal microbiota in the ODNcap-mediated effects. Collectively, our findings suggest that daily oral ingestion of ODNcap may provide preventive effects on allergic bronchopulmonary insults *via* regulation of mechanisms involved in the gut-lung connection.

## Introduction

Asthma is a respiratory syndrome characterized by airway hyperresponsiveness (AHR), inflammation, and remodeling (1). Worldwide, asthma causes about 461,000 deaths annually, and the number of patients with asthma is currently estimated to be about 262 million (2); this number is expected to increase to about 400 million by 2025 (3). Therefore, new and effective alternatives to available drugs such as inhaled corticosteroids are needed to prevent and treat asthma.

Adequate exposure to bacterial components such as endotoxins and DNA through pattern recognition receptors (PRRs) may reduce the risk of atopy and asthma development in childhood by activating host innate immunity (4, 5). Based on this idea (the hygiene hypothesis), asthma therapeutics targeting Toll-like receptors (TLRs), a kind of PRR, have been investigated (6). Synthetic oligodeoxynucleotides (ODNs) containing the unmethylated CpG motif (CpG-ODNs) are TLR9 agonists (7). Previous studies using allergic asthma models have shown that CpG-ODNs suppress type 2 T helper cell (Th2)-mediated airway inflammation and dysfunction primarily through the induction of type 1 T helper cell (Th1) or regulatory-type immune responses (6). For example, Sabatel et al. found that intranasal exposure to CpG-ODNs induced the accumulation of regulatory lung interstitial macrophages and helped prevent inflammatory insults within the airway (8). The clinical efficacy of CpG-ODN-based therapy has been reported in adult patients with mild-to-moderate persistent allergic asthma (9). These findings strongly suggest that CpG-ODNs have promise as nucleic acid therapeutic agents for the prevention of asthma.

Although CpG-ODNs exert outstanding immunological functionality that may provide beneficial effects in the prevention and treatment of allergic asthma, their value as an oral formulation remains unclear. In 2015, we developed a new material (named ODNcap) to realize the oral delivery of ODNs, including CpG-ODNs. ODNcap, which is a nano- to micro-sized carbonate apatite-based ODN-embedded particle, exhibits excellent protection of ODNs against artificial gastric juice, DNase, and autoclaving (10). In addition, we have shown that orally administered ODNcap reaches the intestinal mucosa, where these particles are taken up by macrophages residing in the Peyer’s patches, subsequently triggering immune responses in mice. Therefore, we postulated that ODNcap is an attractive new candidate for the development of a CpG-ODN-based oral formulation for the treatment of allergic asthma.

In this study, we evaluated the preventive effect of free feeding of ODNcap-containing feed (ODNcap-F) on the pathologies of an ovalbumin (OVA)-induced mouse model of allergic asthma. We choose free feeding to deliver ODNcap orally because this method does not cause pain or stress to the animals and supports the concept of “edible CpG-ODNs”. We also explored the mechanism(s) of action of ODNcap feeding using omics-driven approaches.

## Materials and Methods

### Oligodeoxynucleotides

Endotoxin-free, desalted, and phosphorothioated class-B CpG-ODNs [MsST (11)] were synthesized by GeneDesign, Inc. (Osaka, Japan). MsST was reconstituted in endotoxin-free water and sterilized by passage through a 0.22-μm pore microfilter. The sequence of MsST was as follows: 5′-CAGGACGTTGTATCACTGAA-3′.

### Preparation of ODNcap-F and Cap-F

MsST encapsulated in carbonate apatite-based particles was prepared using a modification of a previously established method (10, 12, 13). First, 1 M CaCl_2_ (900 μL) was mixed with 30 mL of inorganic solution (44 mM NaHCO_3_, 0.9 mM NaH_2_PO_4_, 25 mM D-glucose, pH 7.5) containing 6 mg MsST, and the mixture was allowed to stand for 60 min at 37°C. The suspension then was centrifuged at 700 g for 5 min, and the pellet was washed and freeze-dried. The control particles (Cap) were prepared without ODN by the same procedure. Finally, the pellet (1 mg) was blended with a commercial mouse powder feed (1 g, MF, Oriental Yeast, Tokyo, Japan) to prepare ODNcap-feed (ODNcap-F) or Cap-feed (Cap-F).

### Ethics and Procedures for the *In Vivo* Experiments

All experimental procedures were carried out in accordance with the Regulations for Animal Experimentation of Shinshu University, and the animal protocol was approved by the Committee for Animal Experiments of Shinshu University (No. 280063).

Female BALB/c mice (4 weeks of age) were purchased from Japan SLC (Shizuoka, Japan), housed under temperature- and light-controlled conditions, and fed a standard diet (MF, Oriental Yeast) and sterile water *ad libitum*. After preliminary housing for 2 weeks, we established the following four experimental groups (*n* = 6/experiment): non-treatment (NT), standard diet-feed (Ctrl-F), Cap-F, and ODNcap-F. Three independent experiments (Exp. 1 to 3) were performed.

**Figure 1:**
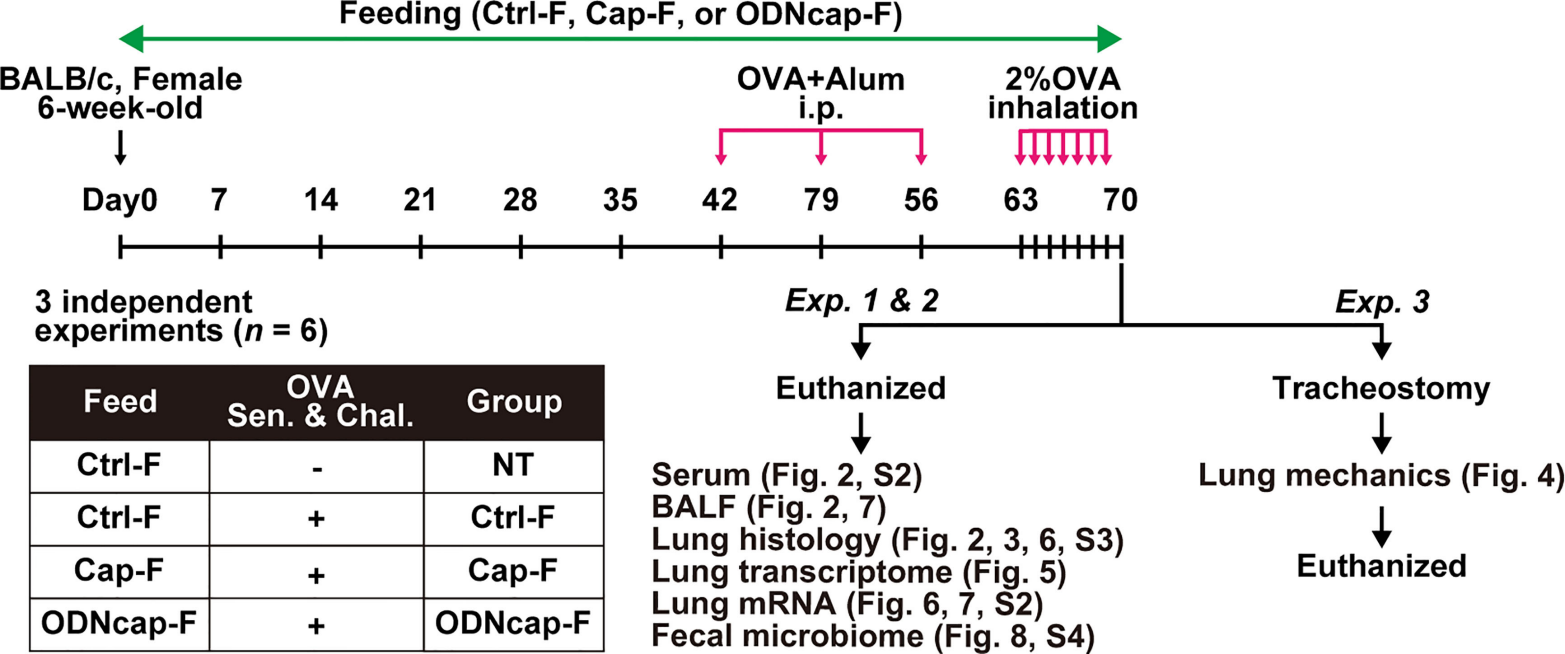
Schematic protocol for the *in vivo* experiments. OVA, ovalbumin; Alum, aluminum oxide gel; i.p., intraperitoneal injection; Sen. & Chal., sensitization and challenge; Ctrl-F, standard feed; Cap-F, control particle-containing feed; ODNcap-F, CpG-oligodeoxynucleotide particle-containing feed; Exp., experiment; BALF, bronchoalveolar lavage fluid.

The experimental protocol is shown schematically in **Figure 1**. Briefly, mice were fed the standard or customized feeds freely for 70 days. An OVA-induced allergic airway inflammation model was used by modification of a previously established method (14). Mice were immunized on Days 42, 49, and 56 with OVA (Sigma-Aldrich, St. Louis, MO, USA) by intraperitoneal injection of 1 μg OVA adsorbed to 2 mg of aluminum oxide gel. From Days 63 to 69, mice were exposed (once daily for 30 min/day) to an aerosol of 2% OVA in phosphate-buffered saline; exposure employed an ultrasonic nebulizer (NE-U17; OMRON, Kyoto, Japan). On Day 70, 24 h after the last OVA inhalation, mice were subjected to different procedures as follows: in Exp. 1 and 2, mice were euthanized to collect serum, feces, bronchoalveolar lavage fluid (BALF), and lungs; in Exp. 3, mice were tracheostomized under anesthesia to measure the lung mechanics.

### Enzyme-Linked Immunosorbent Assay

Levels of OVA-specific IgG_1_ and cytokines in serum and BALF were quantified using commercial enzyme-linked immunosorbent assay (ELISA) kits (IgG_1_: Cayman Chemical, Ann Arbor, MI, USA; cytokines: eBioscience, San Diego, CA, USA) according to the manufacturers’ instructions. Quantification of OVA-specific IgG_2a_ was performed according to a previously established procedure (15).

### Real-Time Quantitative PCR

Total RNA was isolated from the lungs with TRIzol Reagent (Life Technologies, Carlsbad, CA, USA), and then was reverse-transcribed using PrimeScript RT Master Mix (Takara Bio, Shiga, Japan) according to the manufacturers’ instructions. Real-time quantitative PCR was performed with a Thermal Cycler Dice Real Time System II (TaKaRa Bio) using TB Green Premix Ex Taq II (TaKaRa Bio) as described previously (16). The optimized primers for target-specific amplification were purchased from Perfect Real Time Support System (TaKaRa Bio), except for *Il22ra1-* (17), *Reg3g-* (18), and *Extl3*-specific (17) primers that were synthesized by Integrated DNA Technologies (Coralville, IA, USA).

### Flow Cytometric Analysis

**Figure 2:**
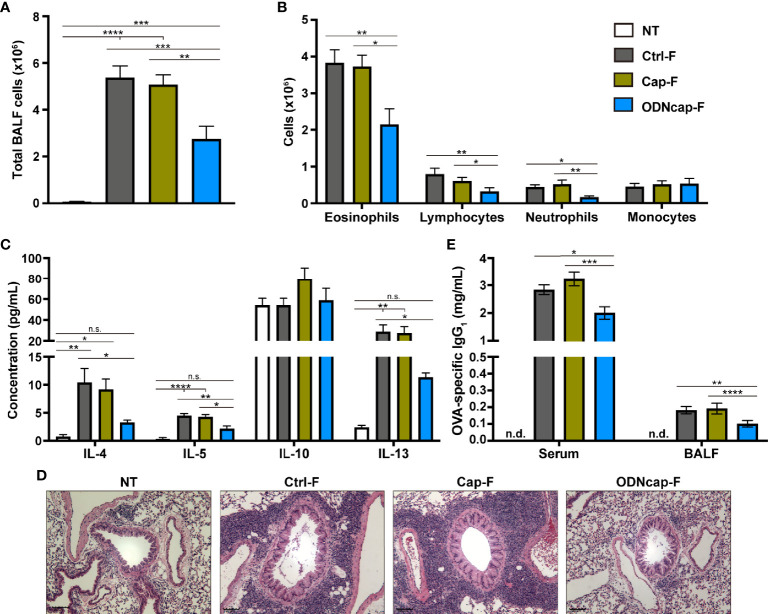
ODNcap-F attenuates allergic airway inflammation. Bronchoalveolar lavage fluid (BALF), serum, and lung sections were prepared on Day 70. **(A)** Total BALF cells were counted by trypan blue staining (*n* = 12). **(B)** Flow cytometric analysis was performed to determine the proportions of eosinophils, neutrophils, lymphocytes, and monocytes in BALF (*n* = 12). **(C)** BALF levels of cytokines were measured by enzyme-linked immunosorbent assay (*n* = 6). **(D)** Representative images of hematoxylin-eosin-stained sections (*n* = 6). Scale bars, 100 μm. **(E)** Serum and BALF levels of ovalbumin (OVA)-specific immunoglobulin G_1_ (IgG_1_) were measured by enzyme-linked immunosorbent assay (*n* = 12). n.d., not detected. Data are shown as the mean ± standard error of the mean. ****P < 0.0001; ***P < 0.001; **P < 0.01; *P < 0.05; n.s., not significant by two-tailed one-way analysis of variance and *post hoc* Tukey’s tests.

The number of BALF cells was counted by trypan blue staining. We omitted the NT group from this analysis because the total number of BALF cells in that group was excessively low (< 1×10^5^ cells; **Figure 2A**). BALF cells were fixed in 4% paraformaldehyde. The fixed cells (1×10^5^ cells/tube) then were stained with fluorescein isothiocyanate (FITC)-conjugated rat anti-mouse CD11b (1/1,000; 101205; BioLegend, San Diego, CA, USA), FITC-conjugated rat anti-mouse Ly6G (1/100; 127606; BioLegend), Alexa Fluor 488-conjugated rat anti-mouse CD3 (1/100; 100210; BioLegend), Alexa Fluor 488-conjugated rat anti-mouse CD19 (1/100; 115521; BioLegend), Alexa Fluor 488-conjugated mouse anti-mouse NK1.1 (1/100; 108718; BioLegend), phycobiliprotein (PE)-conjugated rat anti-mouse SiglecF (1/100; 552126; BD Biosciences, Franklin Lakes, NJ, USA), PE/Cyanine5-conjugated rat anti-mouse CD11b (1/100; 101210; BioLegend), and/or peridinin-chlorophyll-protein-conjugated hamster anti-mouse CD11c (1/200; 117326; BioLegend) antibodies for 60 min at 4°C. The cells then were washed, and the numbers of eosinophils (CD11b^+^ CD11c^–^ SiglecF^+^), neutrophils (CD11b^+^ Ly6G^+^), lymphocytes (CD3^+^ CD19^+^ NK1.1^+^), and monocytes (CD11c^+^) were determined using a flow cytometer (FACSCalibur, BD Biosciences). All data were analyzed using FlowJo software (ver. 10.3; FlowJo, LLC, Ashland, OR, USA).

### Histopathology and Immunohistochemistry

Lungs were fixed in 10% buffered formalin, embedded in paraffin wax, and sectioned (5 μm) according to standard procedures. Hematoxylin and eosin (HE) or periodic acid-Schiff (PAS) staining of the sections was performed according to standard procedures (10, 19). Histopathologic evaluation was performed on the HE-stained sections. PAS-stained sections were used to assess goblet cell hyperplasia. A ranking system (0: none, 1: rare, 2: mild, 3: moderate, 4: intermediate between moderate and gross, 5: gross, and 6: very gross) was employed to score the PAS-positive area around the bronchioles. Immunohistochemistry was performed to detect pulmonary vein cardiomyocytes (PVCs). The deparaffinized sections were subjected to antigen retrieval by microwave irradiation in 10 mM citrate buffer for 10 min. Endogenous alkaline phosphatases and non-specific absorption of antibodies were blocked with Endogenous Peroxidase and Alkaline Phosphatase Blocking Solution (Vector Laboratories, Burlingame, CA, USA) and Blocking One Histo (Nacalai Tesque), respectively. The sections then were reacted with mouse anti-cardiac muscle troponin T (cTnT) (1/40; MA5-12960; Thermo Fisher Scientific) or mouse isotype control (1/1,000; 31903; Thermo Fisher Scientific) antibodies overnight at 4°C. The reaction was detected with ImmPRESS-AP Horse Anti-Mouse IgG Polymer Reagent (Vector Laboratories) and alkaline phosphatase substrate solution (Vector Laboratories) according to the manufacturer’s instructions. All sections were observed by light microscopy (BZ-X800; KEYENCE, Osaka, Japan).

### Assessment of Airway Hyperresponsiveness to Methacholine

Mice were tracheostomized under anesthesia and connected to a ventilator (flexiVent; SCIREQ, Montreal, Canada) to assess responsiveness to methacholine according to a previously established procedure (20). Mice were then exposed to nebulized methacholine (0, 3, 10, and 30 mg/mL), and respiratory system resistance (Rrs) values were monitored after each methacholine challenge using the snapshot technique. All data were analyzed using the flexiWare software (ver. 7.6).

### Lung Transcriptome Profiling

Total RNA was isolated from the lungs of eight mice (two mice/group). Transcriptome profiling based on DNA microarray analysis was performed by Macrogen Japan (Kyoto, Japan) using the SurePrint G3 Mouse Gene Expression 8x60 K platform (Agilent, Santa Clara, CA, USA). The microarray results were extracted using Agilent Feature Extraction software (ver. 11.0) (Agilent), and then log-transformed and normalized using the quantile method. A total of 32,312 probes were identified in the filtered data. The statistical significance of the expression data was determined using the local-pooled-error test and fold change. The false discovery rate (FDR) was controlled by adjusting the *P* values using the Benjamini-Hochberg algorithm. All data analyses were conducted using R software (ver. 3.2.1) (21).

### Western Blotting

BALF samples were boiled in an equal volume of 2× sample buffer (Wako, Osaka, Japan) for 5 min. A 20-μL aliquot was resolved by sodium dodecyl sulfate-polyacrylamide gel (15% polyacrylamide) electrophoresis. The resolved bands were transferred from the gel onto a polyvinylidene difluoride membrane (GE Healthcare, Amersham, UK). The membrane was blocked with 3% skim milk for 60 min at room temperature. The blots then were reacted with rabbit anti-regenerating islet-derived protein 3 gamma (Reg3γ) antibody (1/500; SAB4301004; Sigma-Aldrich) overnight at 4°C, and were further incubated with horseradish peroxidase-conjugated donkey anti-rabbit IgG antibody (1/5,000; 406401; BioLegend) for 60 min at room temperature. The reaction was detected with a lumino-image analyzer (ImageQuant LAS 500; GE Healthcare) with ECL Prime Western Blotting Detection Reagent (GE Healthcare). Densitometric analysis of the signals was performed with ImageJ software (ver. 1.53a) (22).

### 16S Ribosomal RNA Gene Amplicon Sequencing

DNA was isolated from fecal samples using the QIAamp Fast DNA Stool Mini Kit (Qiagen, Hilden, Germany) according to the manufacturer’s instructions. The 16S V3–V4 region of the fecal DNA was amplified as described previously (23, 24). The amplicon was barcoded and enriched by PCR using the Nextera XT Index Kit (Illumina, San Diego, CA, USA), and then purified and quantified. A pool containing equivalent quantities of the barcoded V3–V4 amplicons from each sample was sequenced (by TaKaRa Bio) using a MiSeq instrument (Illumina) to generate paired-end (2×250 bp) Illumina-sequencing data. The QIIME2 pipeline (ver. 2019.1) (25) was employed to process and analyze the data. The demultiplexed raw sequences were filtered by trimming primer sequences, removing low-quality and chimeric reads, joining paired-end reads, and identifying and enumerating amplicon sequence variants (ASVs) using the q2-dada2 plugin. The resultant data were aligned to build a phylogenetic tree using the q2-phylogeny plugin. The q2-diversity plugin was used to compute and statistically analyze the α and β diversity metrics of the aligned data. ASVs were referred to a trained classifier (gg-13-8-99-nb-classifier.qza) using the q2-feature-classifier plugin to assign taxonomy. The denoised data contained 3,991,059 high-quality reads from 48 samples (median: 87,864 reads/sample). The data of three samples that were identified as having excessively low read numbers were abrogated. The data were then rarefied to 55,682 reads per sample in the diversity analyses.

### Statistical Analysis

The statistical analyses of the microarray data and the diversity metrics of the fecal microbiome are described in the subsections titled “Lung transcriptome profiling” and “16S ribosomal RNA gene amplicon sequencing”, respectively. Other statistical analyses were performed using a statistical software package (GraphPad Prism 8; GraphPad Software, San Diego, CA, USA). Two-tailed one-way analysis of variance followed by Tukey’s tests or the Kruskal–Wallis test with Dunn’s multiple comparisons was used to determine the significance of the differences. Differences between groups in the lung mechanics data were analyzed using a mixed effects model for repeated measures; the *P* values were adjusted using an FDR method. Correlations between the relative abundance of fecal bacteria and OVA-specific IgG_1_ levels were identified using Spearman’s correlation. Differences at *P* < 0.05 were considered significant.

## Results

### ODNcap-F Attenuates Allergic Airway Inflammation

Allergen-induced airway inflammation represents an important pathology in allergic asthma and is mediated primarily by the Th2 immune response (1). Therefore, we investigated the effect of free feeding of ODNcap-F on the prevention of OVA-induced airway inflammation in mice. The body weight and feed consumption of each mouse were comparable among all groups over the course of the study (**Supplementary Figure 1**). Signatures of Th2-mediated inflammation in BALF, lungs, and serum were analyzed on Day 70. The total number of BALF cells was increased similarly in Ctrl-F and Cap-F compared to the non-treated healthy control group (NT) (**Figure 2A**). This increase was suppressed significantly in ODNcap-F (**Figure 2A**). Flow cytometric analysis of BALF cells showed a significant decrease in the number of eosinophils, neutrophils, and lymphocytes in ODNcap-F compared to Ctrl-F and Cap-F (**Figure 2B**). Th2 cytokines (interleukin [IL]-4, IL-5, and IL-13) in BALF accumulated to significantly higher levels in Ctrl-F and Cap-F compared to NT, whereas the levels of these cytokines were comparable between NT and ODNcap-F (**Figure 2C**). In histopathological analysis with HE-stained lung sections, similar patterns of massive infiltration of inflammatory cells around the bronchi were observed in Ctrl-F and Cap-F, but were suppressed in ODNcap-F (**Figure 2D**). OVA-specific immunoglobulin G_1_ (IgG_1_) was detected in the serum and BALF of OVA-sensitized/challenged mice, while the level of these antibodies was significantly reduced in ODNcap-F compared to Ctrl-F and Cap-F (**Figure 2E**). Collectively, these results showed that free feeding of ODNcap-F prophylactically suppresses OVA-induced Th2 responses and inflammation.

Previous reports have demonstrated that CpG-ODNs strongly induce the Th1 immune response to counteract to the Th2-biased response (26–28). Therefore, we measured levels of OVA-specific IgG_2a_ in the serum and interferon-gamma (IFNγ) in BALF, as well as the expression of IFNγ-encoding (*Ifng*) mRNA in the lungs, as markers of Th1-mediated responses. Unexpectedly, the serum level of OVA-specific IgG_2a_ was comparable among OVA-sensitized/challenged groups (**Supplementary Figure 2A**). The BALF level of IFNγ was under the detection limit in all mice (data not shown). *Ifng* expression in the lungs was significantly upregulated in Ctrl-F and Cap-F compared with NT, whereas the *Ifng* transcript level in ODNcap-F resembled the baseline level (**Supplementary Figure 2B**). The levels in BALF of IL-10, a key mediator of the regulatory-type immune response (29), were comparable among all groups (**Figure 2C**). These results suggested that the attenuation of allergic airway inflammation by ODNcap-F feeding may not be involved in inducing the protective Th1- and IL-10-mediated counter-immunity.

### ODNcap-F Attenuates Bronchial Goblet Cell Hyperplasia

**Figure 3:**
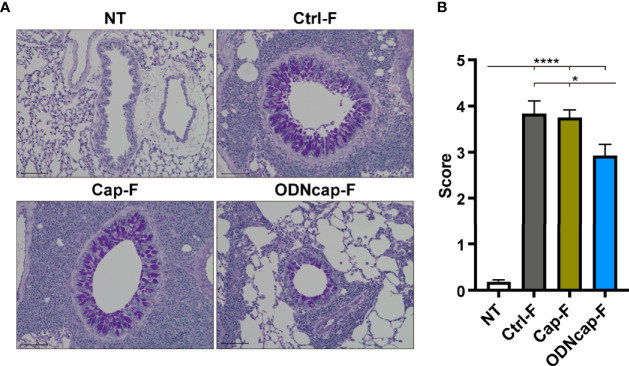
ODNcap-F attenuates bronchial goblet cell hyperplasia. Lung sections were prepared on Day 70. **(A)** Representative images of periodic acid-Schiff (PAS)-stained sections (*n* = 6). Scale bars, 100 μm. **(B)** The PAS-positive area around the bronchi was scored using the ranking system described in the Materials and Methods (*n* = 6). Six to eight bronchi per animal were assessed to obtain the average individual score. Data are shown as the mean ± standard error of the mean. ****P < 0.0001; *P < 0.05 by two-tailed one-way analysis of variance and *post hoc* Tukey’s tests.

Airway remodeling accompanied by goblet cell hyperplasia in airway epithelia also contributes to the pathogenesis of asthma (1). We observed PAS-stained lung sections to evaluate mucin production from goblet cells in the bronchi (**Figure 3**). PAS-positive cells in the bronchial epithelia were prominent in Ctrl-F and Cap-F, but were significantly decreased in ODNcap-F (**Figure 3**). These results showed that free feeding of ODNcap-F attenuates the development of goblet cell hyperplasia and mucus hyperproduction in the airways of OVA-sensitized/challenged mice.

### ODNcap-F Attenuates Airway Hyperresponsiveness

**Figure 4:**
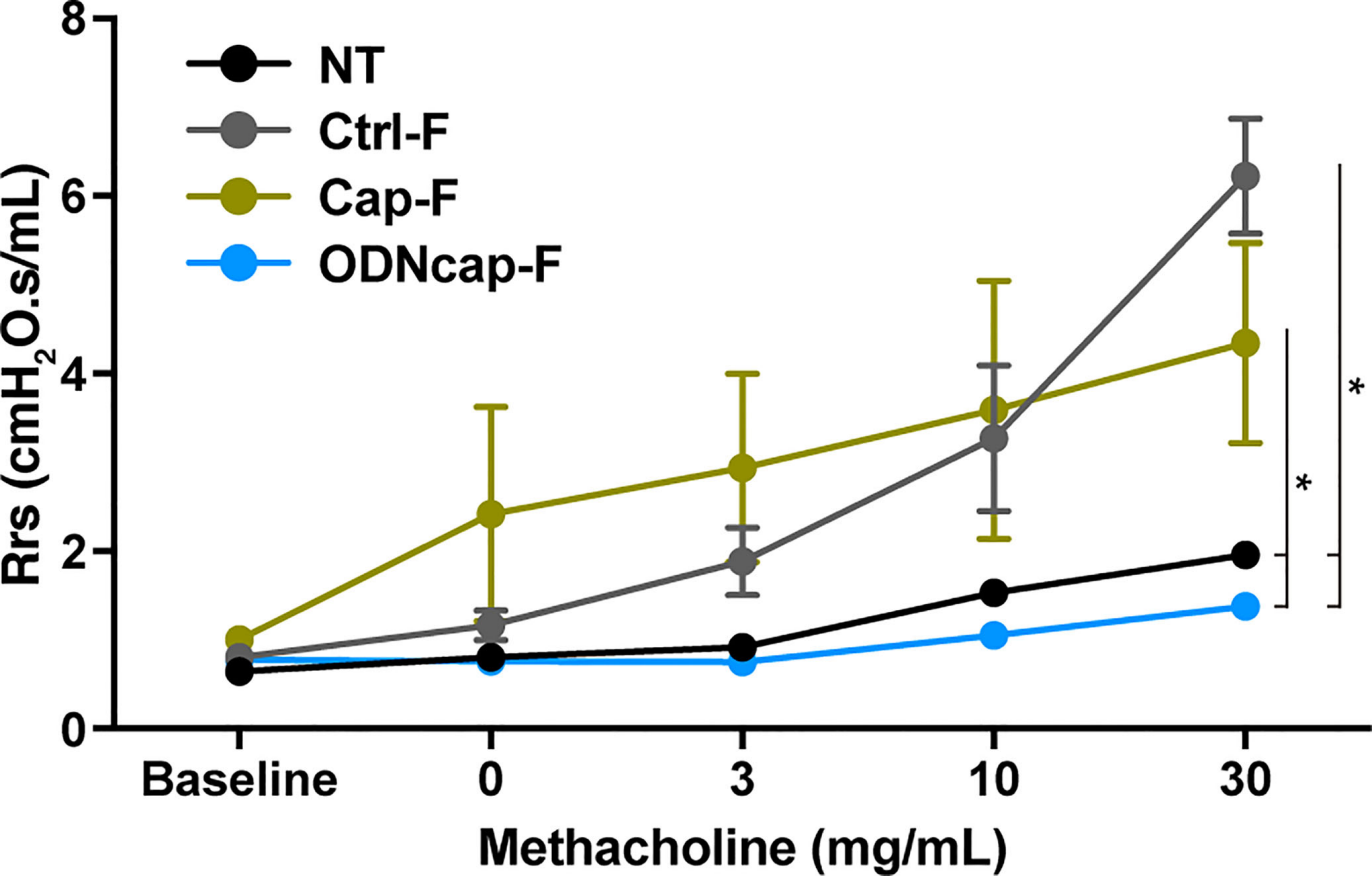
ODNcap-F attenuates airway responsiveness to methacholine. On Day 70, anesthetized mice were connected to a ventilator and then sequentially exposed to various inhaled concentrations of methacholine (0 to 30 mg/mL). Measurements of respiratory system resistance (Rrs) were monitored (*n* = 6). Data are shown as the mean ± standard error of the mean. *P < 0.05 by mixed effects model for repeated measures. The *P* values were adjusted using an FDR method.

To investigate the effect of free feeding of ODNcap-F on AHR, we evaluated airway responsiveness to methacholine challenge using the forced oscillation technique. The values of respiratory system resistance (Rrs) (**Figure 4**) in Ctrl-F were increased by methacholine challenges, and these effects were dose dependent. The values of Rrs in Cap-F were comparable to those in Ctrl-F. The Rrs values in ODNcap-F remained at baseline even with methacholine challenge. These results showed that free feeding of ODNcap-F attenuates OVA-induced AHR.

### ODNcap-F Alters the Lung Transcriptome in OVA-Sensitized/Challenged Mice

**Figure 5:**
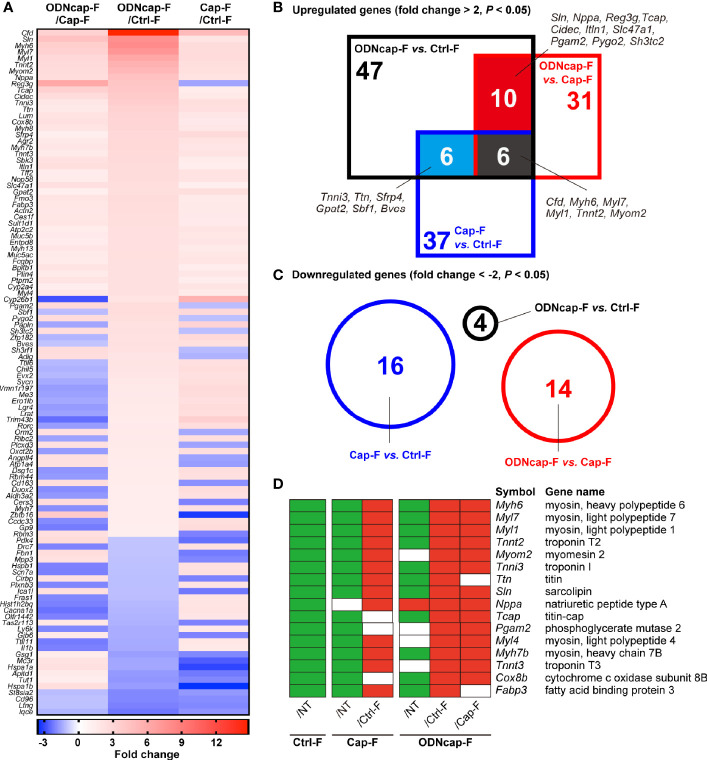
Transcriptomic profiling in the lungs. Lungs were collected on Day 70. Total RNA from the lungs was subjected to DNA microarray analysis (*n* = 2). **(A)** A heatmap shows 108 genes that were differentially expressed among the OVA-sensitized/challenged groups (*P* < 0.05 by local-pooled-error tests and |fold change| > 2). **(B, C)** Venn diagrams show significantly upregulated **(B)** and downregulated **(C)** genes (*P* < 0.05 by local-pooled-error tests and |fold change| > 2). **(D)** Panels visualize differences in the expression of cardiac muscle-related genes among all experimental groups. Green and red boxes represent significantly downregulated and upregulated expression, respectively (*P* < 0.05 by local-pooled-error tests). No significant differences are indicated as blank (white) boxes. The *P* values were adjusted using an FDR method.

To elucidate the suppressive mechanism(s) of action of ODNcap-F in OVA-induced airway insults, we analyzed the lung transcriptome using DNA microarray analysis. Although this analysis was conducted with few replicates (two mice/group), we identified 108 genes that were differentially expressed among the OVA-sensitized/challenged groups (**Figure 5A**). Significantly upregulated and downregulated genes are visualized in **Figures 5B, C**, respectively. Interestingly, gene ontology analysis showed that genes related to cardiac muscle contraction (GO:0060048; *Myh6*, *Myl4*, *Myl1*, *Tcap*, *Tnni3*, *Tnnt2*, *Ttn*, *Nppa*, and *Tnnt3*) were significantly upregulated in ODNcap-F compared to Ctrl-F, Cap-F, or both. Further analyses revealed that genes preferentially or highly expressed in cardiac muscle [specifically, *Myl7* (30), *Myom2* (31), *Sln* (32), *Pgam2* (33), *Cox8b* (34), *Myh7b* (35), and *Fabp3* (36)] also were upregulated in ODNcap-F (**Figures 5A, B, D**). In contrast, the levels of all of these transcripts were decreased in Ctrl-F compared to NT (**Figure 5D**). Among the genes exhibiting ODNcap-F-specific upregulation, we identified *Reg3g*, which encodes the antimicrobial peptide Reg3γ (**Figure 5A**).

### ODNcap-F Inhibits Allergic Injury of Pulmonary Vein Cardiomyocytes

**Figure 6:**
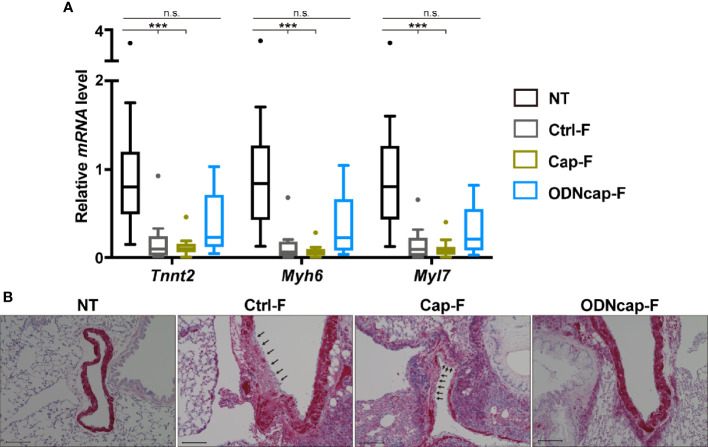
ODNcap-F protects pulmonary vein cardiomyocytes from allergic injury. Lung total RNA and sections were prepared on Day 70. **(A)** Expression levels of cardiac muscle-specific genes in the lung were measured by real-time quantitative PCR (*n* = 12). Data are shown as Tukey’s box plots. ***P < 0.001; n.s., not significant by Kruskal–Wallis test with Dunn’s multiple comparisons. **(B)** Lung sections prepared on Day 70 were stained with anti-cardiac muscle troponin T (cTnT) antibody (*n* = 6). Representative images are shown. Positive reaction of the antibody (red) was observed around the large pulmonary veins. Injured areas (arrows) of the cTnT-positive cells were observed in Ctrl-F and Cap-F, but not in NT or ODNcap-F. Scale bars, 100 μm.

Cardiomyocytes, components of cardiac muscle, are known to be present in the lungs, specifically along the large pulmonary veins (37). Using quantitative PCR, we confirmed that mRNA expression of cardiomyocyte-specific markers (specifically, *Tnnt2*, *Myh6*, and *Myl7*) in the lungs was significantly suppressed in Ctrl-F and Cap-F compared to NT, whereas the expression of these genes in ODNcap-F was comparable to that in NT (**Figure 6A**). Lung sections were stained for cTnT to confirm the presence of pulmonary vein cardiomyocytes (PVCs). In NT, staining for cTnT constituted a dense ring around the large pulmonary vein (**Figure 6B** and **Supplementary Figure 3**). Interestingly, a disjunct area in the cTnT-positive ring was observed occasionally in Ctrl-F as well as Cap-F, suggesting allergic injury of PVCs (**Figure 6B**). Notably, such clearly injured areas were not observed in ODNcap-F (**Figure 6B**). These results suggested that free feeding of ODNcap-F inhibits allergic injury of PVCs.

### ODNcap-F Promotes Secretion of Reg3γ to the Airway

**Figure 7:**
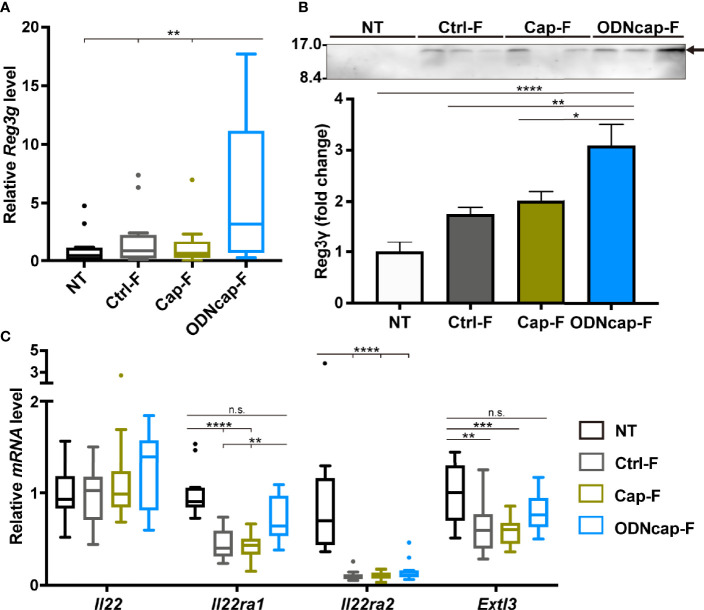
ODNcap-F induces Reg3γ in the lungs. Lung total RNA and bronchoalveolar lavage fluid (BALF) were prepared on Day 70. **(A, C)** Expression levels of indicated genes in the lung were measured by real-time quantitative PCR (*n* = 12). Data are shown as Tukey’s box plots. ****P < 0.0001; ***P < 0.001; **P < 0.01; n.s., not significant by two-tailed one-way analysis of variance (ANOVA) and *post hoc* Tukey’s tests. **(B)** Aliquots (10 μL) of each BALF sample were subjected to western blotting with anti-Reg3γ antibody (*n* = 6). A representative result is shown. Densitometric analysis was performed to quantify the specific bands (arrow). Data are shown as the mean ± standard error of the mean. ****P < 0.0001; **P < 0.01; *P < 0.05 by two-tailed one-way ANOVA and *post hoc* Tukey’s tests.

In lungs, Reg3γ is expressed in airway epithelial cells (AECs) responsive to methicillin-resistant *Staphylococcus aureus* infection or IL-22 (17, 38). Interestingly, a previous study demonstrated that Reg3γ induced by IL-22 inhibits allergic airway inflammation in asthma models (17). To confirm that free feeding of ODNcap-F promotes Reg3γ expression in the lung, we analyzed *Reg3g* expression in the lungs and secretion of Reg3γ into BALF. The *Reg3g* transcript level was significantly upregulated in ODNcap-F compared to that in other groups (**Figure 7A**). Secretion of Reg3γ into BALF also was increased in ODNcap-F (**Figure 7B**). We next analyzed the lung mRNA expression of genes encoding IL-22 signaling-related proteins, which would be expected to contribute to the induction of Reg3γ production. The expression of *Il22ra1*, which encodes a functional IL-22 receptor (IL-22R1) (39), was significantly impaired in Ctrl-F and Cap-F, but was restored in ODNcap-F (**Figure 7C**). The expression levels of *Il22* and *Il22ra2*, which encode a natural antagonist of IL-22 signaling (IL-22 binding protein) (39), were comparable among the allergen-sensitized/challenged groups (**Figure 7C**). We also found that the expression of *Extl3*, which encodes a putative receptor for Reg3γ (exostatin-like 3; EXTL3) (40), was significantly downregulated in Ctrl-F and Cap-F compared to NT, whereas *Extl3* expression was comparable between NT and ODNcap-F (**Figure 7C**). Elsewhere, it has been reported that IL-22R1 and EXTL3 are preferentially expressed on AECs in the lungs (17). Collectively, these results suggested that free feeding of ODNcap-F regulates the mechanisms for the induction, secretion, and action of Reg3γ in AECs to suppress allergic airway inflammation.

### ODNcap-F Causes a Change in theFecal Microbiome

**Figure 8:**
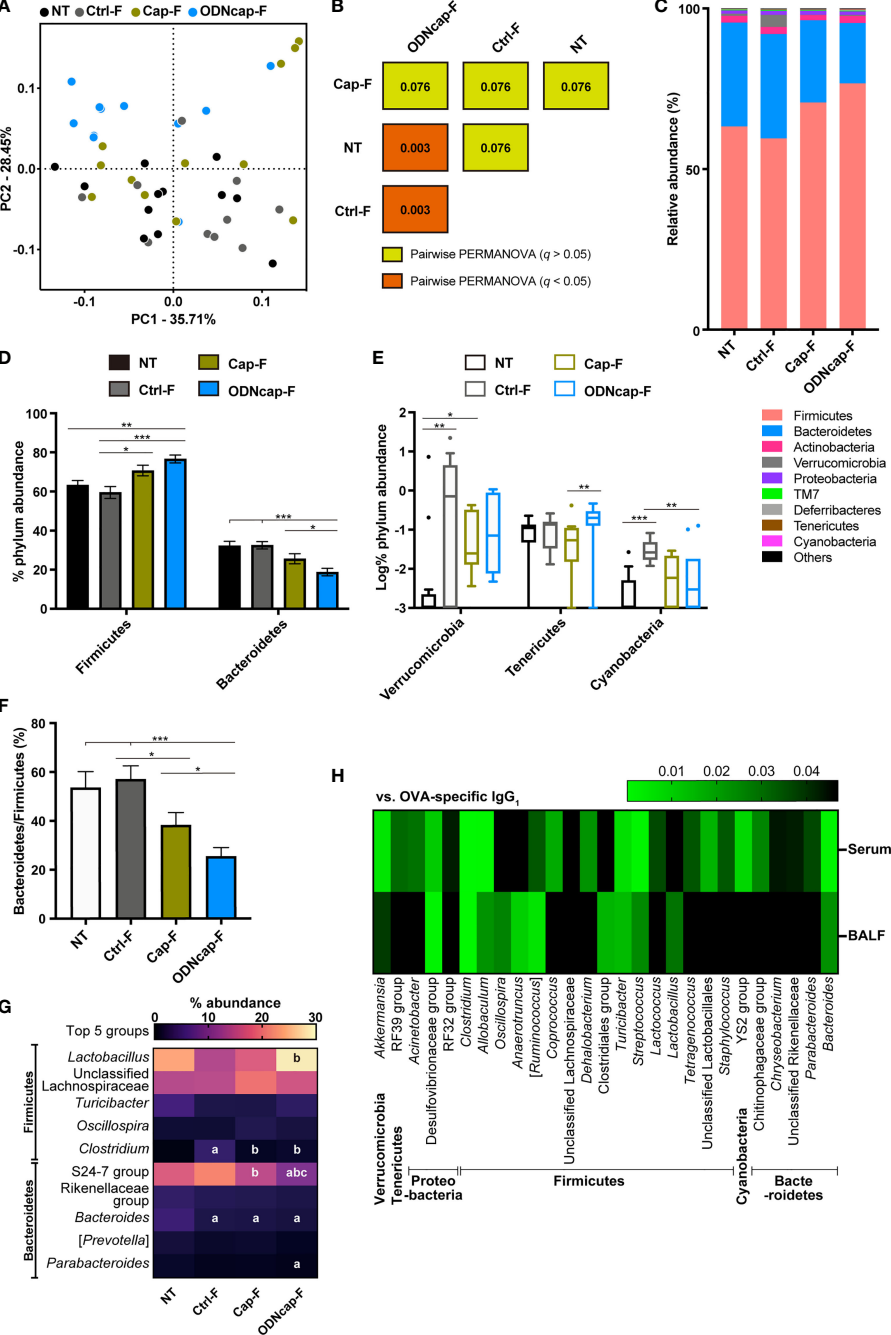
ODNcap-F alters the structure and composition of the fecal microbiota. Feces were collected on Day 70. The V3–V4 regions of 16S rRNA genes were amplified from fecal DNA and subjected to next-generation sequencing (*n* = 12). The data were processed and analyzed using the QIIME2 pipeline. **(A)** Two-dimensional plot of the weighted UniFrac-based principal coordinate analysis of the microbiome. **(B)** Results of pairwise permutational multivariate analysis of variance (PERMANOVA) based on weighted UniFrac distances are visualized. Values in the boxes indicate *q*, which is an adjusted *P* value. **(C–E)** Phylum-level composition (proportional abundance) of the microbiota in each group. **(F)** Bacteroidetes to Firmicutes ratio. **(C)** Bars on the graph indicate means. **(D, F)** Data are shown as the mean ± standard error of the mean. ***P < 0.001; **P < 0.01; *P < 0.05 by two-tailed one-way analysis of variance (ANOVA) and *post hoc* Tukey’s tests. **(E)** Data are shown as Tukey’s box plots. ***P < 0.001; **P < 0.01; *P < 0.05 by two-tailed Kruskal–Wallis test with *post hoc* Dunn’s multiple comparisons. **(G)** Heatmap showing the mean values of proportional-relative abundance of the top five groups belonging to the Bacteroidetes or Firmicutes phylum. Letters in the boxes represent significant differences; a, *vs.* NT; b, *vs.* Ctrl-F; c, *vs.* Cap-F (*P* < 0.05 by two-tailed one-way ANOVA and *post hoc* Tukey’s tests). **(H)** Heatmap shows *P*-values of Spearman’s correlation between ovalbumin-specific immunoglobulin G_1_ (IgG_1_) levels in serum or bronchoalveolar lavage fluid (BALF) and the proportional relative abundance of the fecal bacterial groups.

To evaluate the effects of ODNcap-F on the gut microbiota, next-generation sequencing data for fecal 16S rRNA gene amplicons were analyzed. α diversity indices were comparable among all groups (**Supplementary Figure 4**). Principal coordinate analysis (**Figure 8A**) and permutational multivariate analysis of variance (**Figure 8B**) based on weighted UniFrac distances revealed that the fecal microbiome structure of ODNcap-F was significantly different from those of NT and Ctrl-F. Changes in the abundances of Firmicutes and Bacteroidetes (the two most-abundant phyla) resulted in a significant decrease in the ratio of Bacteroidetes to Firmicutes in ODNcap-F compared to those of other groups (**Figures 8C, D, F**). Other phyla with minor abundances were also altered in the gut microbiome of ODNcap-F (**Figure 8E**). In deeper analysis within Firmicutes and Bacteroidetes phyla, the proportions of some bacterial groups, such as the S24-7 family and *Clostridium* genus, in ODNcap-F were significantly different from those in other groups (**Figure 8G**). To identify specific gut bacteria related to allergic inflammation, Spearman’s correlations between the proportions of each bacterial group and the amounts of OVA-specific IgG_1_ in serum and BALF were analyzed. We found 27 bacteria groups that significantly correlated with OVA-specific IgG_1_ levels in serum, BALF, or both (**Figure 8H**).

## Discussion

The benefits of parenteral CpG-ODN injection for the prevention and treatment of various disorders, including asthma, have been established in numerous animal and clinical studies (6, 41). However, the long-term and continuous administration of such injections to healthy persons is not suitable for the goal of disease prevention. In this context, the development of a CpG-ODN-based oral formulation that can efficiently trigger immune responses against disease would be desirable (42, 43). Here, we prepared customized feed containing ODNcap, which is an acid-resistant CpG-ODN particle. We demonstrated, using an asthma model, that free feeding of ODNcap-F for 70 days attenuated eosinophilia and Th2 cytokine production in the airways, allergen-specific IgG_1_ production in serum and BALF, bronchial goblet cell hyperplasia, and AHR to methacholine. These findings suggest that the daily oral intake of ODNcap acts prophylactically to attenuate allergic airway inflammation and asthmatic symptoms.

Kitagaki et al. found that the short-term intragastric administration of CpG-ODNs afforded prophylactic and therapeutic effects on allergen sensitization and airway eosinophilia in an asthma model (44). However, those authors also found that a relatively high dose (1,000 μg/mouse) was needed to optimize the treatment with oral CpG-ODNs. The high dose of CpG-ODNs may increase the risk of severe side effects (45) and pharmaceutical prices. In the present study, mice consumed (per day per mouse) 1.5–2.5 g of ODNcap-F, containing 1.5–2.5 mg of ODNcap. Given that the ODN encapsulation rate of ODNcap is approximately 1% (10), we estimated that the mice ingested 15–25 μg of CpG-ODNs per day per animal. This dosage is comparable to the effective dose (5–100 μg/mouse) of parenteral administration (6).

The potential ability of CpG-ODNs to induce Th1-biased or immunoregulatory responses may help reduce the Th2-mediated pathology of asthma (6). In the present study, although we showed that ODNcap-F attenuated the Th2 responses in the lungs of allergen-sensitized/challenged mice, we did not find any signatures of activation of Th1- and regulatory-type responses in BALF, lung transcripts, or serum of the ODNcap-F group on Day 70. These data strongly suggested that ODNcap-F employs other mechanisms to attenuate Th2-mediated airway inflammation, at least in the challenge phase. To seek those other mechanisms, we comprehensively analyzed the lung transcriptome and the fecal microbiome of ODNcap-fed mice.

Surprisingly, our small-scale lung transcriptomic analysis revealed that ODNcap-F restored expression of cardiac muscle-related genes encoding myosin chains, troponins, titin, and so on, that otherwise were decreased by the allergen challenge. Gene expression of cardiac-specific markers (e.g., *Tnnt2*, *Myh6*, and *Myl7*) clearly was reduced in the lungs of Ctrl-F and Cap-F, suggesting the loss of PVCs. As expected, immunohistochemical analysis revealed the partial elimination of PVCs wrapping the large pulmonary veins in the lungs of Ctrl-F and Cap-F. This report is the first to demonstrate that allergic inflammation is associated with the injury of PVCs. Although the role of PVCs in lung physiology and pathology remains largely unknown, recent studies have suggested the involvement of these cells in respiratory disorders including asthma. For example, genome-wide association studies have identified cardiac-related genes, including *CTNNA3* (encoding α-T-catenin) and *TTN* (encoding titin), as a factor for the pathogenesis or exacerbation of asthma (46–48). Folmsbee et al. reported that *Ctnna3*-knockout mice have altered susceptibility to chemical- and allergen-induced AHR, and suggested that PVCs contribute to the shaping of the inflammatory milieu of adjacent airways (49, 50). Moreover, Yee et al. reported that neonatal hyperoxia, which induces pulmonary hypertension and reduces lifespan, causes depletion in PVCs in adult mice (51). These facts led us to hypothesize that ODNcap-F protects PVCs, thereby suppressing allergic insults in the lungs.

We also discovered that free feeding of ODNcap-F promotes the secretion of Reg3γ to the airway under conditions of allergen challenge. A previous study in a house dust mite (HDM)-induced asthma model demonstrated that allergic airway inflammation and AHR were exacerbated by neutralization of allergen challenge-induced Reg3γ production in the airway (17). This observation implies that ODNcap-F-induced upregulation of airway Reg3γ likely contributes to the suppression of airway inflammation in the challenge phase. Previous studies have pointed to possible roles for IL-22 and EXTL3 in the induction or action of Reg3γ. Namely, Ito et al. showed that IL-22 induces the production of Reg3γ in AECs, cells that express a functional IL-22R-encoding gene (*Il22ra1*) to high levels, in a signal transducer and activator of transcription 3-dependent manner (17). In addition, those authors reported that *Extl3* was preferentially expressed in AECs in the lung, and that intratracheal administration of recombinant Reg3γ suppressed HDM-induced type 2 innate immune responses in the lung. Accumulating evidence indicates that Reg3γ acts like a hormone through binding to EXTL3 (52–55). In the present study, we found that the expression level of *Il22ra2*, which encodes an IL-22 antagonist, but not that of *Il22*, was altered in the lungs of allergen-sensitized/challenged mice, suggesting that IL-22 activity was indirectly enhanced in the challenge phase. Importantly, we also showed that lung levels of *Il22ra1* and *Extl3* transcripts were downregulated in Ctrl-F and Cap-F, while the expressions of those genes were maintained in ODNcap-F. These results suggest that ODNcap-F regulates or protects the receptivity of AECs to both IL-22 and Reg3γ in the IL-22-activated condition.

In previous studies, we and another group showed that orally administered ODNcap primes mucosal immunity in the gut (10, 56), a modality that fundamentally differs from parenteral applications of CpG-ODNs that target systemic immunity. In addition, the priming site of ODNcap may differ from that of orally administered “naked” CpG-ODNs, given that DNA delivered orally is absorbed in the small intestine and mainly primes systemic sites such as the spleen (57, 58). This unique characteristic of ODNcap implies the possibility that ODNcap-F modulates the gut ecosystem, which is shaped by complex interactions between gut-associated microorganisms and host cells, to attenuate airway insults. Barcik et al. reported that microbial dysbiosis in the gut ecosystem may contribute to the onset and aggravation of asthma (59). We therefore used a 16S metagenomic technique to analyze the fecal microbiome at the end point of the experiment. β diversity analyses revealed that free feeding of ODNcap-F for 70 days caused a structural change in the fecal microbiome, regardless of the allergen challenge. The change in β diversity in the ODNcap-F group was attributed primarily to a decrease of the Bacteroidetes/Firmicutes ratio. Bacteria belonging to those two phyla represent the majority of the mouse and human gut microbiota and produce various bioactive molecules such as short-chain fatty acids and lipopolysaccharides. Interestingly, an increased abundance of *Bacteroides* species, a main group of the Bacteroidetes phylum in humans, has been found in infants with a high risk of developing atopy and autoimmunity (60, 61). It has also been reported that high-level exposure to *Bacteroides* lipopolysaccharides contributes to the onset of type 1 diabetes (61). Conversely, *Lactobacillus* species, which were enriched in the ODNcap-F group, have been found to be decreased in allergic children (62) and to provide beneficial effects toward the prevention of allergies (63). Therefore, alteration of the gut microbiota by free feeding of ODNcap-F may be a preferred condition for the attenuation of allergic airway inflammation.

In conclusion, free feeding of a customized feed containing ODNcap preventively attenuated allergic airway inflammation and AHR in an OVA-induced mouse model of asthma. As CpG-ODNs have shown promise in phase II clinical trials (but failed in phase III), our finding may be valuable for the development of a new prophylactic agent for allergic asthma and a cost-effective oral formulation based on CpG-ODNs. However, in order to facilitate the clinical translation of ODNcap, further basic research is required to validate its efficacy, safety, therapeutic potential, and precise mechanism of action in more realistic models of allergic asthma than the OVA-induced model (e.g., HDM-induced models). Interestingly, our omics analyses and follow-up experiments indicated the participation of PVCs, airway Reg3γ, and gut microbiota in the ODNcap-F-mediated effects. These results imply that ODNcap-F regulates the mechanisms involved in the gut-lung connection to suppress allergic bronchopulmonary insults. Future comprehensive studies to test this hypothesis will bring new insights into the pathophysiology and therapeutics of asthma.

## Data Availability Statement

The datasets presented in this study can be found in online repositories. The names of the repository/repositories and accession number(s) can be found below: DDBJ Sequence Read Archive: DRA012444 and NCBI Gene Expression Omnibus: GSE180287.

## Ethics Statement

The animal study was reviewed and approved by Committee for Animal Experiments, Shinshu University.

## Author Contributions

TOk, SS, FN, TOg, and TSa conducted the experiments. TOk and SS performed the mathematical analyses. SS and TSh wrote the paper. SS and TSh conceived of and designed the work. TSh supervised the work. All authors contributed to the article and approved the submitted version.

## Funding

This study was supported by JSPS KAKENHI Grant Nos. 17H03907 and 20H03125, and by a grant from the LOTTE Foundation, Kobayashi Foundation, and Urakami Foundation for Food and Food Culture Promotion to TSh.

## Conflict of Interest

The authors declare that the research was conducted in the absence of any commercial or financial relationships that could be construed as a potential conflict of interest.

## Publisher’s Note

All claims expressed in this article are solely those of the authors and do not necessarily represent those of their affiliated organizations, or those of the publisher, the editors and the reviewers. Any product that may be evaluated in this article, or claim that may be made by its manufacturer, is not guaranteed or endorsed by the publisher.
