# Supplementary material for: Free Feeding of CpG-Oligodeoxynucleotide Particles Prophylactically Attenuates Allergic Airway Inflammation and Hyperresponsiveness in Mice
Source: Front Immunol. 2021 Nov 19;12:738041. doi: 10.3389/fimmu.2021.738041 (PMC8639529; doi:10.3389/fimmu.2021.738041)
Supplement: Supplementary file 1 [file DataSheet_1.docx]

Supplementary Material

# Supplementary Figures

**Supplementary Figure 1.** Changes in the body weight (A) and feed intake (B) of mice during the experiment. (A) No significant differences (*P* ≥ 0.05 by two-tailed one-way analysis of variance with post hoc Tukey’s test) in body weight were seen in any week among the groups (*n* = 12). (B) The mice consumed from 1.5 to 2.5 g of feed per mouse per day (*n* = 12). Data are shown as the mean ± standard error of the mean.

**Supplementary Figure 2.** Measurements of the levels of OVA-specific IgG_2a_ (in serum) and *Ifng* expression (in lung tissue). Serum and lung total RNA were prepared on Day 70. (A) Serum levels of OVA-specific IgG_2a_ were analyzed by ELISA (*n* = 12). (B) *Ifng* expression in the lung was measured by real-time quantitative PCR (*n* = 12). Data are shown as the mean ± standard error of the mean. *, *P* < 0.05; n.s., not significant (*P* ≥ 0.05) by two-tailed one-way analysis of variance with post hoc Tukey’s test. A450, absorbance at 450 nm.

**Supplementary Figure 3.** Immunohistochemistry of cTnT in lung sections. Lung sections prepared on Day 70 were stained with anti-cTnT or mouse isotype control antibodies (*n* = 6). Representative images from NT are displayed. Scale bars = 100 μm.

**Supplementary Figure 4.** Analyses of the α diversity of the fecal microbiota. Feces were collected on Day 70. The V3–V4 region of the 16S rRNA gene was amplified from fecal DNA and subjected to next-generation sequencing (*n* = 12). Data were processed and analyzed using the QIIME2 pipeline. α diversity indices (Shannon’s diversity index, observed OTUs, and Faith’s phylogenic diversity) were comparable among all groups (*P* ≥ 0.05 by the Kruskal–Wallis test). Data are shown as Tukey’s box plots. OTUs, operational taxonomic units.
